# Towards Whole Body Fatigue Assessment of Human Movement: A Fatigue-Tracking System Based on Combined sEMG and Accelerometer Signals

**DOI:** 10.3390/s140202052

**Published:** 2014-01-27

**Authors:** Haiwei Dong, Izaskun Ugalde, Nadia Figueroa, Abdulmotaleb El Saddik

**Affiliations:** 1 School of Electrical Engineering and Computer Science, University of Ottawa, 800 King Edward, K1N 6N5, Ottawa, ON, Canada; E-Mail: elsaddik@uottawa.ca; 2 Division of Engineering, New York University Abu Dhabi, P.O. Box 129188, Abu Dhabi, UAE; E-Mail: izaskun.ugalde@nyu.edu; 3 Learning Algorithms and Systems Laboratory, Ecole Polytechnique Federale de Lausanne (EPFL), Station 9, CH 1015, Lausanne, Switzerland; E-Mail: nadia.figueroafernandez@epfl.ch

**Keywords:** localized muscular fatigue, fatigue information fusion, wireless wearable system

## Abstract

This paper proposes a method to assess the overall fatigue of human body movement. First of all, according to previous research regarding localized muscular fatigue, a linear relation is assumed between the mean frequency and the muscular working time when the muscle is experiencing fatigue. This assumption is verified with a rigorous statistical analysis. Based on this proven linearity, localized muscular fatigue is simplified as a linear model. Furthermore, localized muscular fatigue is considered a dynamic process and, hence, the localized fatigue levels are tracked by updating the parameters with the most current surface electromyogram (sEMG) measurements. Finally, an overall fatigue level is computed by fusing localized muscular fatigue levels. The developed fatigue-tracking system is evaluated with two fatigue experiments (in which 10 male subjects and seven female subjects participated), including holding self-weight (dip start position training) and lifting weight with one arm (arm curl training).

## 1. Introduction

In the field of biomechanics, fatigue is defined as a decrease in physical movement performance due to internal and external forces [1]. Cumulative physical fatigue can lead to musculoskeletal disorders (MSD) [2]. Thus, monitoring and tracking fatigue is of great importance in order to prevent the development of such disorders. Many applications benefit from fatigue monitoring, such as promoting muscle performance and growth in sports training [3]; preventing intolerant exercise in rehabilitation [4,5], *etc.* In this paper, we propose a method to assess the overall fatigue status of human movement and verify the proposed method by a prototype. An extensive body of literature exists on the subject of monitoring localized muscular fatigue, thus our proposed method is based on this research. Our final objective is to provide a solution to the assessment of human fatigue statuses in whole body movements. In this paper, we focus on developing a prototype of a wearable fatigue-tracking system to quantify overall fatigue in a specific human movement.

Existing approaches to the monitoring of muscular fatigue can be categorized into two types: simulation-based and experiment-based. Regarding the simulation-based methods, numerous muscular fatigue models have been built according to the Ca^2+^ cross-bridge mechanism [6,7], force-PH relation [8,9], elastic element modeling (e.g., Hill's model) [10], *etc.* However, for experiment-based methods, the use of surface electromyogram (sEMG), a non-invasive technique, has become popular in clinical fatigue measurement, as the subject experiences minimal discomfort while measuring fatigue levels (no needle punctures are required) [11,12]. Studies from the field of kinesiology have shown that the power spectrum variables (including mean frequency, median frequency, and mode frequency) [13] of the sEMG signal decrease during sustained contraction. In practice, the mean frequency of the sEMG signal has been widely used for detecting muscular fatigue due to its low sensitivity to noise [14,15]. Several computational methods for calculating the mean frequency from the power spectrum have been introduced in literature, including classical methods (e.g., the periodogram, and the Blackman-Tukey estimator) and modern parametric model methods (such as autoregressive, moving average, autoregressive moving average, *etc.*) [16].

Since our method and prototype must be functional in practical applications, we adopt the experiment-based methods. Specifically, we use the mean frequency to indicate localized muscular fatigue due to its low sensitivity to noise [6]. This paper targets at the quantification and monitoring of the overall fatigue status of human body movement based on localized muscular fatigues. Furthermore, we treat “overall fatigue” as a dynamic process and, thus, it is updated by the most current sEMG measurements. The following issues are addressed in detail in this paper:

*Quantifying an overall fatigue assessment corresponding with a human movement*: The current literature regarding fatigue addresses the problem either at the muscle level [17] or at the joint level [18]. Fatigue assessment at the muscle level is commonly considered fatigue classification. Different classifiers can be applied, such as the neural network (NN) classifier, the Bayesian classifier, the fuzzy logic (FL) classifier, the support vector machine (SVM) classifier, and the hidden Markov model (HMM) classifier [19]. On the other hand, the fatigue assessment at the joint level is usually simplified to a first-order differential process of the current joint load torque divided by the maximum joint load [18,20]. In this paper, we address on assessing “overall fatigue level” corresponding to a specific human movement. More specifically, we fuse the muscle fatigue levels by weighted average, where the weight defined here is customized case by case due to different applications. As an example in our research, the weight is set as a normalized gradient of the muscular fatigue level.

*Tracking fatigue status*: In previous research, muscular fatigue is essentially considered a static process, as it is assessed only by the current measurements [3,12]. However, fatigue is naturally a dynamic process, meaning that a previous fatigue status also influences a current fatigue status. Thus, we introduce a “forgetting factor”, whose physical meaning is the amount of previous fatigue status information that should be considered [21]. In this paper, we propose a tracking method of muscular fatigue based on the concept of dynamically “forgetting previous fatigue information”. In computation, as the current muscular fatigue status is iteratively updated by the fatigue information at the previous time step and most current measurement, it has low sensitivity to disturbances.

This paper is organized as follows: in Section 2, we describe the system architecture, basic scheme, experimental setting and signal pre-possessing; in Section 3, we illustrate segmentation and connection of the sEMG signals by detecting periodic movements; in Section 4, we describe the statistical analysis and simplification of the localized muscular fatigue level; in Section 5, we present the tracking scheme of the localized muscular fatigue levels and their fusion to generate an overall fatigue level; in Section 6, the verification of our proposed method is illustrated by the fatigue tracking performance in two fatigue experiments; and in Section 7, we conclude the paper and provide plans for our future work.

## 2. System Architecture and Experiment Setting

### 2.1. Hardware

**Figure 1.**
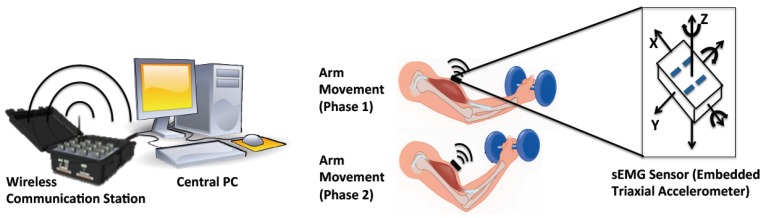
System architecture of the fatigue-tracking system. The system consists of a central PC, a wireless communication station, and sEMG sensors.

The fatigue-tracking system includes a central processing PC, a wireless communication center and a couple of sEMG sensors (Figure 1). The sEMG sensor used is a Delsys Trigno wireless sensor (37 mm × 26 mm × 15 mm, 16-bit resolution, 2,000 Hz sampling rate), which consists of a parallel-bar-based EMG measurement device and a triaxial accelerometer. The triaxial accelerometer is used to capture dynamic movements and impact simultaneously with the sEMG data measurements.

As the sEMG sensor is required to be placed at the center of the muscle during the muscle's contraction, the body movement can be monitored by an accelerometer. The wireless communication center (maximum communication distance is 40 m) is used to receive the online data from the sEMG sensors and send it to the central processing PC. It can simultaneously communicate with 16 sEMG sensors (*i.e.*, having 16 EMG channels, 48 accelerometer channels). The central PC computes and displays the tracked fatigue level by human interactive interface.

### 2.2. Basic Scheme

**Figure 2.**
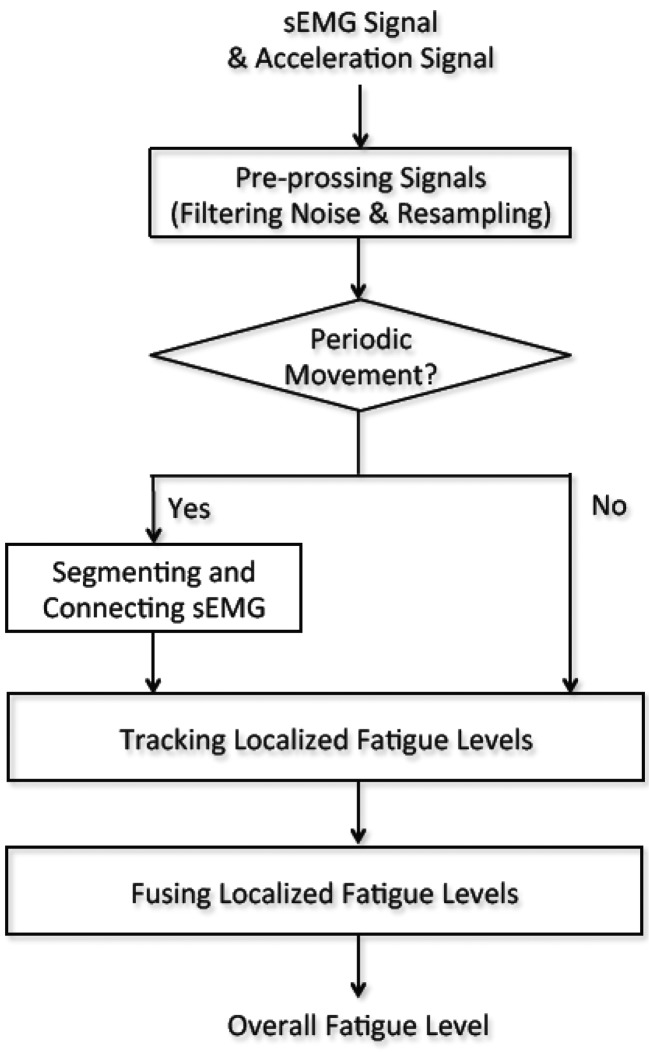
Scheme diagram of the system. Periodic movement is recognized by analyzing the acceleration signal pattern. Non-active sEMG signals are cut off by segmenting the original sEMG signal. The processed sEMG signal is used for tracking localized muscular fatigue levels. The overall fatigue level is obtained by fusing the localized fatigue levels.

The sEMG signal processing is performed in a central PC and illustrated in Figure 2. Initially, the sEMG signal and corresponding acceleration signal are filtered to remove high frequency noise. Next, the two signals are re-sampled so that both signals have the same sampling rates. The filtered acceleration signal is then used to recognize periodic movements. If periodic movements are detected, the filtered sEMG signal is segmented and connected to form a new sEMG signal for mean frequency calculation. The localized fatigue levels are then tracked by updating the parameters with the most current sEMG measurement. If there is no periodic movement recognized, the sEMG segmentation and connection procedure is skipped. Finally, the overall fatigue level is computed by fusing the localized fatigue levels. The details of this method are outlined in Sections 3–5.

### 2.3. Experimental Procedures

**Figure 3.**
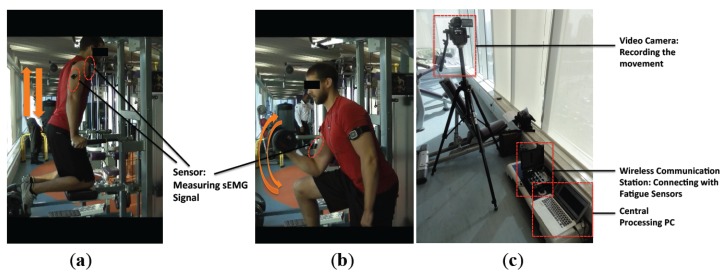
Fatigue experiment settings. (**a**) In Experiment 1, the subject was asked to hold their self-body weight by arms (dip start position training). (**b**) In Experiment 2, the subject was asked to repeatedly lift up the weight with their right arm (arm curl training). (**c**) The experiment equipment included a video camera, a wireless communication station, and a central processing PC.

We conducted two experiments for the verification of our method (Figure 3). The experiment procedures have been approved by the university's committee. In Experiment 1, the subjects are asked to hold their self-body weight with both arms for a period of time (dip start position training); in Experiment 2, the subjects were asked to periodically lift a weight (10 kg for males, and 6 kg for females) with their right arm (arm curl training). In total, 17 subjects, including 10 males and 7 females, were studied (mean ± SD): age 30.47 ± 6 years; body mass 71.71 ± 16.81 kg; body height 172.82 ± 11.25 cm; and body mass index (BMI) 23.86 ± 4.03 kg/m^2^. Before initiating the experiment, the subjects were briefed with the experiment's purpose and procedures. The subjects were asked to attach the sEMG sensors on the following muscle groups: biceps brachii, anterior deltoids, and triceps brachii. Taking into account the muscles measured in the experiments, shaving body hair were not necessary. The skin of each subject was cleaned with 90% alcohol and then the sensors were attached using double faced adhesive tape (based on the instructions of the Delsys sensors).

After warming up, each subject was required to hold their self-body weight for 1 min in Experiment 1 (Figure 3a) where the angle between lower arm and upper arm is approximately 120°, rest for 5–10 min, and then periodically lift a weight (complete bending-stretching movement) with the right arm approximately every 2.5 s during a 1 min time span in Experiment 2 (Figure 3b). Lifting pace was roughly informed by rhythmic sound. Here, the maximum duration of 1 min was determined due to the arm-shaking phenomenon that appeared for all the subjects before 1 min, which clearly indicated the subjects' high fatigue status.

During the experiments, the sEMG signals were measured and further transferred to the central processing PC via a wireless communication station where the signal processing was executed in Matlab. The whole experiment process was recorded by a video camera (Figure 3c).

### 2.4. Pre-Processing Signals

The sEMG signal and the acceleration signal are filtered using the Butterworth filter. Specifically, in designing the Butterworth filter, the lowest order of the filter *n* and normalized cutoff frequency *W_n_* are firstly computed by the designed filter parameters, including passband corner frequency *W_p_*, stopband corner frequency *W_s_*, passband ripple *R_p_*, and stopband attenuation *R_s_*. After that, the Butterworth filter is determined by *n* and *W_n_*. According to the feature of sEMG and acceleration signals in our designed fatigue experiments, the filter specifications of the two signals are set as follows: for sEMG signal, *W_p_* = 0.1 Hz, *W_s_* = 0.4 Hz, *R_p_* = 3 dB, *R_s_* = 40 dB; for acceleration signal, *W_p_* = 0.003 Hz, *W_s_* = 0.006 Hz, *R_p_* = 3 dB, *R_s_* = 40 dB. The settings of the moving window are as follows: the window length is 0.125s and the window overlap is 0.063 s. In our system, the sampling rate of the sEMG signal and the acceleration signal is 4,000 Hz and 296 Hz, respectively. To ensure the two signals have the same data length in analysis, the measured sEMG signal is resampled in the rate of 4,000/296.

## 3. Automatic Periodic Movement Detection

**Figure 4.**
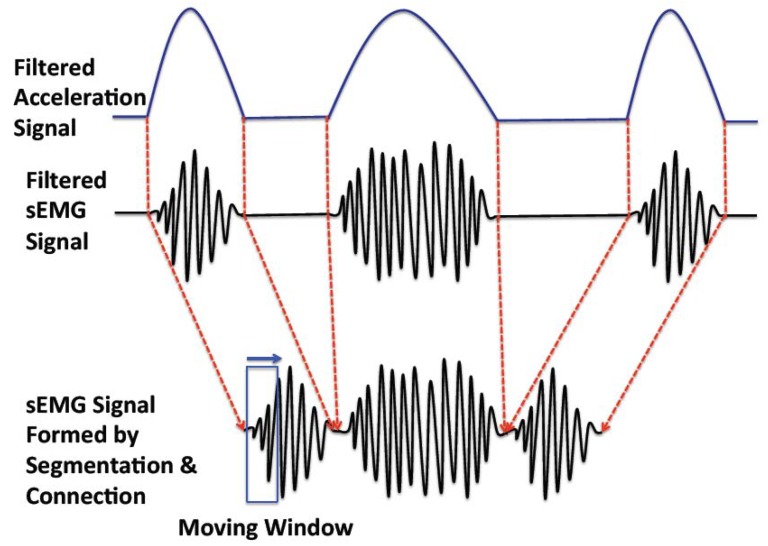
Segmentation and connection of the sEMG signal. The filtered sEMG signal is segmented based on the periodic movement pattern. The active sEMG signal parts are connected to form a new sEMG signal for the following moving window calculation.

There are two working patterns in muscle movement: sustained contraction (considered as non-periodic movement) and alternate contraction-recovery (considered as periodic movement) [12]. The former is simpler to analyze, as it is a continuous and consistent movement pattern; the latter is more complex, as it consists of a contraction and a recovery phase, corresponding with active sEMG and inactive sEMG signals, respectively. To assess the muscular fatigue of the alternate contraction-recovery muscle movement, we segment the contraction movement and connect the corresponding active sEMG signals (Figure 4).

Although the periodic movement pattern can possibly be detected by the sEMG signal, this pattern is much clearer when the acceleration signal is used. In the following part, we apply correlation analysis on the acceleration signal in order to detect the periodic movement. In detail, we use the cross-covariance to analyze the acceleration signal to detect if the recorded movement is a periodic movement and, if so, to find out the breaking points for segmentation.

In detail, for the acceleration signal *ACC* with *N* samples, we compute the cross-covariance *ϕ_ACC_* by [16]:

(1) $$\begin{array}{l} \phi_{\text{ACC}}\left(m\right)=E\left\{\left(\text{ACC}\left(n+m\right)-\mu_{\text{ACC}}\right){\left(\text{ACC}\left(n\right)-\mu_{\text{ACC}}\right)}^{*}\right\}\\ =\{\begin{array}{ll} \overset{N-|m|-1}{\underset{n=0}{\text{∑}}}\left(\text{ACC}\left(n+m\right)-\frac{1}{N}\overset{N-1}{\underset{i=0}{\text{∑}}}\text{ACC}\left(i\right)\right)\cdot \left(\text{AC}C^{*}-\frac{1}{N}\overset{N-1}{\underset{i=0}{\text{∑}}}\text{ACC}{\left(i\right)}^{*}\right) & m\geq 0\\ Cov_{\text{ACC}}^{*}\left(-m\right) & m<0 \end{array} \end{array}$$

where *E*{·} is the expected value operator, *μ_ACC_* is the mean values of *ACC*, and * denotes the complex conjugate. The periodic movement is confirmed if:

(2) $$\frac{\mathrm{Max}\left(\phi_{\text{ACC}},1\right)-\mathrm{Max}\left(\phi_{\text{ACC}},2\right)}{\mathrm{Max}\left(\phi_{\text{ACC}},2\right)}<\varepsilon$$

Otherwise, a non-periodic movement is confirmed. Here, *Max*(*i*,*j*) returns the *j*-th largest value in the vector *i*. *ε* is a threshold determining the periodic movement judge.

## 4. Modeling Localized Fatigue Level

First of all, we define the localized fatigue level as:

(3) $$l_{\text{fatigue}}=\frac{f_{\text{mean},t}}{f_{\text{mean},0}}-1$$

where *f_mean_*_,0_ and *f_mean_*_,_*_i_* are the mean frequency at the initial moment and moment *t*, respectively. Its physical meaning is the percentages of the relative decrease of the mean frequency. Specifically, the mean frequency of the sEMG *f_mean_*_,_*_i_* at the moment *t* is the average frequency of the power spectrum, *i.e.*:

(4) $$f_{\text{mean},t}=\frac{\int_{0}^{\infty }\omega \text{PS}D_{\text{sEMG}}\;\left(\omega \right)d\omega }{\int_{0}^{\infty }\text{PS}D_{\text{sEMG}}\;\left(\omega \right)d\omega }$$

where *PSD_sEMG_*(*ω*) is the power spectrum density of the sEMG signal and *ω* is the frequency variable. In this paper, we compute the power spectrum density of the sEMG signal by fast Fourier transform (FFT), as the power spectrum format is identical to the real part of the FFT, *i.e.*:

(5) $$\text{PS}D_{\text{sEMG}}\;\left(\omega \right)=\text{FF}T_{\text{sEMG}}\;\left(\omega \right)\text{FF}T_{\text{sEMG}}^{*}\;\left(\omega \right)={|\text{FF}T_{\text{sEMG}}\;\left(\omega \right)|}^{2}$$

where *FFT_sEMG_*(*ω*) is the fast Fourier transform of the sEMG signal. $\text{FF}T_{\text{sEMG}}^{*}\;\left(\omega \right)$ is the complex conjugate of *FFT_sEMG_*(*ω*).

According to previous literature on mean frequency for time-series analysis [19], we assume that the mean frequency decreases linearly with working time as the fatigue gradually increases [14,15], *i.e.*:

(6) $$f_{\text{mean},t}=\varphi_{t}\cdot t+\varepsilon_{t}$$

where *φ_t_* is slope parameter of the model. *ε_t_* is remaining term. *t* is working time under fatigue status of the muscle. In the following, we use the measurement in Experiment 1 to statistically prove the linear relation (Equation (6)). At the 95% confidence level (*i.e.*, 5% significance level), we propose a hypothesis for F test on the slope parameter as:

*H_φ_*_0_: *φ_t_* = 0 (meaning that working time is not a useful predictor of mean frequency change)
*H_φ_*_1_: *φ_t_* ≠ 0 (meaning that working time is a useful predictor of mean frequency change)

We apply analysis of variance (ANOVA) on the measurement data of Experiment 1 to prove the above hypotheses in Table 1 where m1 to m10 and f1 to f7 are the subject number in male and female group respectively. *p* is the significance number.

It is shown that all the p values in Table 1 are less than 0.05 and, thus, we reject the null hypothesis *H_φ_*_0_. This means that the variation explained by the linear model is not due to random chance. Meaning that we have evidence to conclude that the slope parameter *φ_t_* is not 0, and hence it can be a predictor of *f_mean_*_,_*_i_*. In other words, considering the individual differences and the above verification, the linear model (Equation (6)) is suitable for describing mean frequency of different individuals. Based on the linear model of the mean frequency in Equation (6), we can simplify the localized fatigue level as:

(7) $$l_{\text{fatigue}}=\frac{\varphi_{t}\cdot t+\varepsilon_{t}}{f_{\text{mean},0}}-1$$

## 5. Tracking and Fusing Localized Muscular Fatigue Levels

According to the statistical analysis outlined in the previous section, *φ_t_* is an adequate predictor of *f_mean_*_,_*_i_*. Thus, we can track the muscular fatigue level *l_fatigue_* by identifying the slope *φ_t_*. As *φ_t_* is a time-varying coefficient, we estimate its value at each moment. Here, the estimation model of the mean frequency can be formed as:

(8) $${\hat{f}}_{\text{mean},t}={\hat{\varphi }}_{t}\cdot t+\varepsilon_{t}$$

where *f̂_mean_*_,_*_t_* is the estimated value of *f_mean_*_,_*_i_*. *φ̂_t_* is the estimated slope parameter. Equation (8) suggests that *f̂_mean_*_,_*_t_* can be updated by estimating the slope parameter *φ̂_t_*. The idea here is to update *φ̂_t_* by using the most current information of the mean frequency. Thus, tracking the localized muscular fatigue level can be formulated as:

(9) $$l_{\text{fatigue}}=\frac{{\hat{\varphi }}_{t}\cdot t+\varepsilon_{t}}{f_{\text{mean},0}}-1$$

Here, *φ̂_t_* can be obtained by minimizing the total error between the real and estimated model in the sense of least-squares, *i.e.*:

(10) $${\hat{\varphi }}_{t}=\arg\min{\int_{0}^{t}e}^{-\int_{r}^{t}\lambda \left(s\right)ds\cdot {\left\|f_{\text{mean},t}-{\hat{\varphi }}_{t}t\right\|}_{2}}dr$$

where 0 ≤ *λ* ≤ 1 is the “forgetting factor” indicating how much the previous information of localized fatigue level is used in calculating the current one. Specifically, *λ* ≡ 0 means previous muscular fatigue information is completely ignored (*i.e.*, only the current measurement is used when calculating the current muscular fatigue level) and *λ* ≡ 1 means all the previous muscular fatigue information is considered in the calculation of the current fatigue level. In order to calculate Equation (10), the specific method we use is “recursive least squares with forgetting” [21,22].

In detail, by defining *p_t_* = *t*^−2^, we can rewrite Equation (10) as:

(11) $${\hat{\varphi }}_{t}={\hat{\varphi }}_{t-1}+\frac{p_{t-1}t}{\lambda +t^{2}\;p_{t-1}}\left(f_{\text{mean},t}-{\hat{\varphi }}_{t-1}t\right)$$

where the updating rule of *p_t_* can be written as:

(12) $$p_{t}=\frac{1}{\lambda }\left(1+\frac{p_{t-1}t^{2}}{\lambda +t^{2}p_{t-1}}\right)\;p_{t-1}$$

With Equations (11) and (12), we can update *φ̂_t_* by using *φ̂_t_*_−1_ and the most current measurement *f_mean_*_,_*_i_*. From the work done by Branch and Evan [23], the least squares with forgetting is a restricted form of the Kalman filter with constant gain equaling to 1− *λ*.

**Figure 5.**
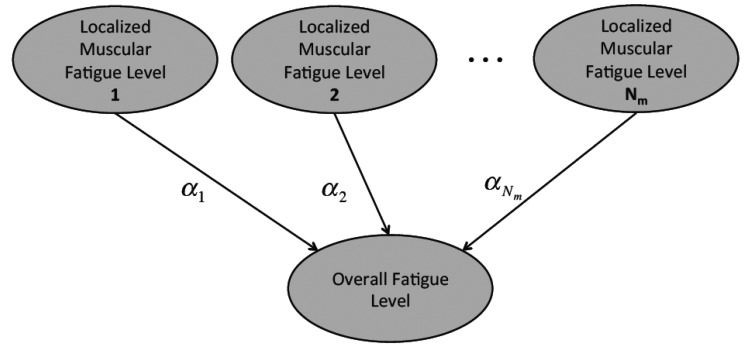
Fusion of localized muscular fatigue levels. Different localized muscular fatigue levels (corresponding with different muscles) are fused together to represent the overall fatigue status of a human movement.

The tracked localized muscular fatigue levels are further fused to define the overall fatigue level of a human body movement (Figure 5) where *N_m_* is the total muscle number and *α_i_* (*i* = 1, 2,…, *N_m_*) is the weight coefficient. In detail, the localized muscular fatigue level fusion can be calculated by:

(13) $$\{\begin{array}{l} L_{\text{fatigue}}=\alpha_{1}l_{\text{fatigue},1}+\alpha_{2}l_{\text{fatigue},2}+\cdots +\alpha_{N_{m}}l_{\text{fatigue},N_{m}}\\ \alpha_{1}+\alpha_{2}+\cdots +\alpha_{N_{m}}=1 \end{array}$$

where *α*_1_, *α*_2_, ⋯, *α_N_m__* are fatigue level fusion coefficients, representing the impact from the localized muscular fatigue level to the overall fatigue level. These coefficients are determined by field experts based on different human movement patterns and application objectives. In this paper, the weight *α_i_* is considered as a normalized gradient of the *i*-th muscle's fatigue level, *i.e.*, meaning that the localized fatigue level with bigger changing rate contributes more in the calculation of the overall fatigue level:

(14) $$\alpha_{i}=\frac{\left\|\nabla l_{\text{fatigue},i}\right\|}{\overset{N_{m}}{\underset{i=1}{\text{∑}}}\left\|\nabla l_{\text{fatigue},i}\right\|}$$

The procedures of overall fatigue level computation are summarized in Algorithm 1 as follows:

**Algorithm 1**: Overall fatigue level computation**Input**: Raw sEMG and corresponding acceleration signals**Output**: Overall fatigue level
1:Filter and resample the input signals;2:For *i* from 1 to *N_m_*3: Calculate the self-covariance of the filtered acceleration signal *ϕ_ACC_*;4: If *ϕ_ACC_* < *ε*, the movement is recognized as a periodic movement. Then, the corresponding sEMG signal is segmented and connected to form a new sEMG signal, otherwise, do nothing;5: Compute the initial mean frequency of the sEMG signal *f_mean_*_,_*_0_*;6: Initialize *λ*, *φ̂_t_* and *p_t_*. *φ̂_t_*(0) can be 0 or an initial guess of the slope parameter, as *p_t_* is defined as *t*^−2^, *p_t_*(0) has to be set as a relatively large number;7: Compute *f_mean_*_,_*_i_* and update *φ̂_t_* and *p_t_* by Equations (11) and (12);8: Calculate the localized fatigue level *l_fatigue,i_* by Equation (9);9:End For10:Compute the overall fatigue level *l_fatigue_* by *l_fatigue,i_* (Equation (13)).


## 6. Results and Discussion

In the following Sections from 6.1 to 6.4, we take one subject's case to discuss the fatigue-tracking results. In Section 6.5, we summarize the outcomes from all the participants and evaluate the developed system.

### 6.1. Measurement

**Figure 6.**
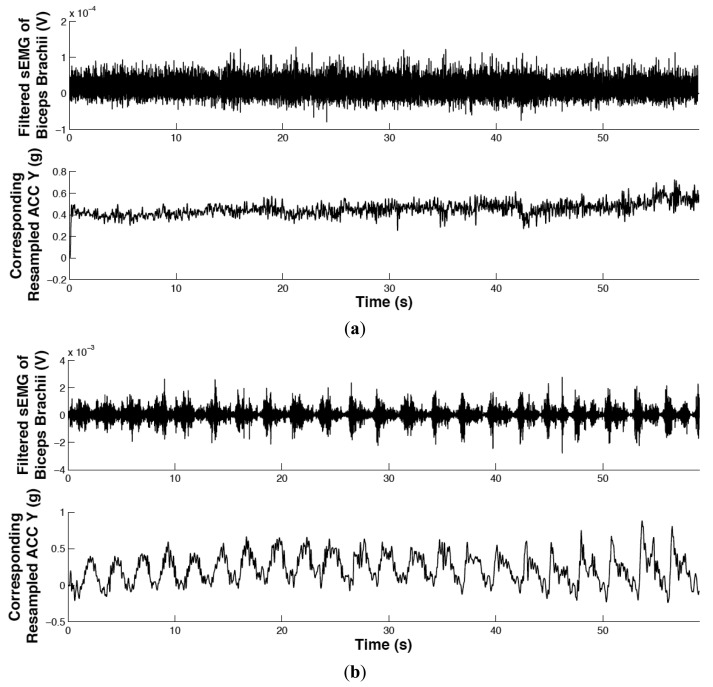
Pre-processed measurements. Filtered sEMG signal of biceps brachii and corresponding resampled rotational acceleration signal in Experiment 1 (**a**) and Experiment 2 (**b**). The movement in Experiment 1 is non-periodic, whereas the movement in Experiment 2 is periodic.

The filtered sEMG signal of biceps brachii and corresponding resampled acceleration signal in Experiments 1 and 2 are shown in Figure 6, where the upper and lower subplots correspond with the sEMG signal and acceleration signal, respectively. The horizontal axis is time (in seconds) and the vertical axes are the sEMG signal (in V.) and acceleration signal (in g) where g ≈ 9.8 m/s^2^. The resolution of the acceleration signal is calculated with 8 bits over the full 3.3 V dynamic range, encompassing accelerometer ideal maximum outputs of ±2.1 g. It is clear that the movement in Experiment 2 is periodic, whereas in Experiment 1 it is non-periodic.

### 6.2. Correlation Analysis

**Figure 7.**
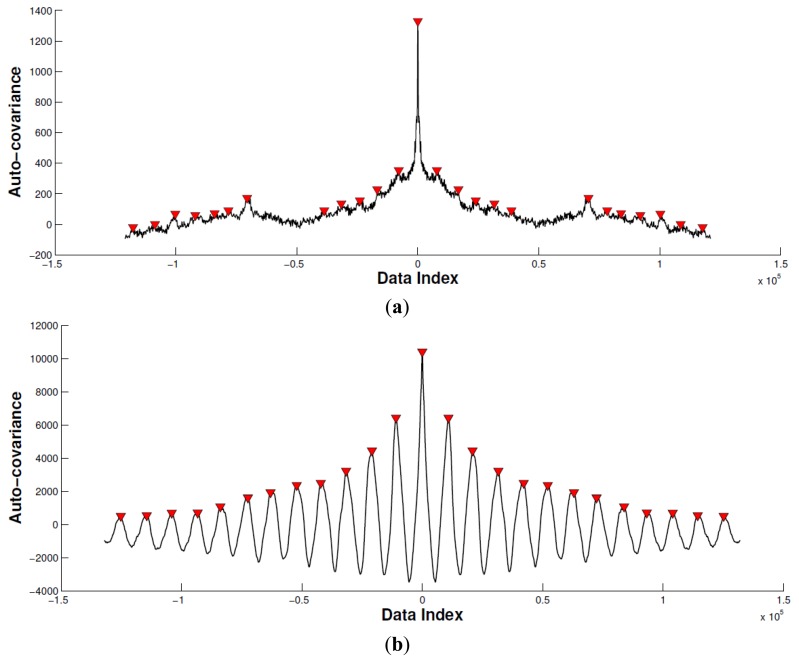
Self-correlation of the acceleration signal corresponding with biceps brachii. (**a**) Experiment 1. (**b**) Experiment 2. In Experiment 1, as the difference between the first and second local peaks is larger than the predefined threshold, the movement pattern is recognized as non-periodic. In contrast, the movement pattern in Experiment 2 is determined as periodic.

In this subsection, we calculate the cross-correlation to analyze the periodic property of the acceleration signal corresponding with biceps brachii (Figure 7). Specifically, we remove the mean of the acceleration signal before computing the cross-correlation. Then, we limit the maximum lag to 50% of the signal to achieve a good estimation of the cross-covariance.

By comparing the peak difference between the largest and second largest local peak with the predefined threshold *ε*, we determine if the movement is periodic. In this paper, the threshold *ε* is set as twice that of the second largest local peak value. As seen of in Figure 7, the horizontal axis indicates data index, and vertical axis indicates the auto-covariance result. The red triangle emphasizes the local peaks. According to the proposed method mentioned, Experiment 1 is recognized as a non-periodic movement, and Experiment 2 is recognized as a periodic movement. Based on this, the sEMG signal in Experiment 2 is segmented and connected as a new sEMG signal for the mean frequency calculation.

### 6.3. Mean Frequency Trend

**Figure 8.**
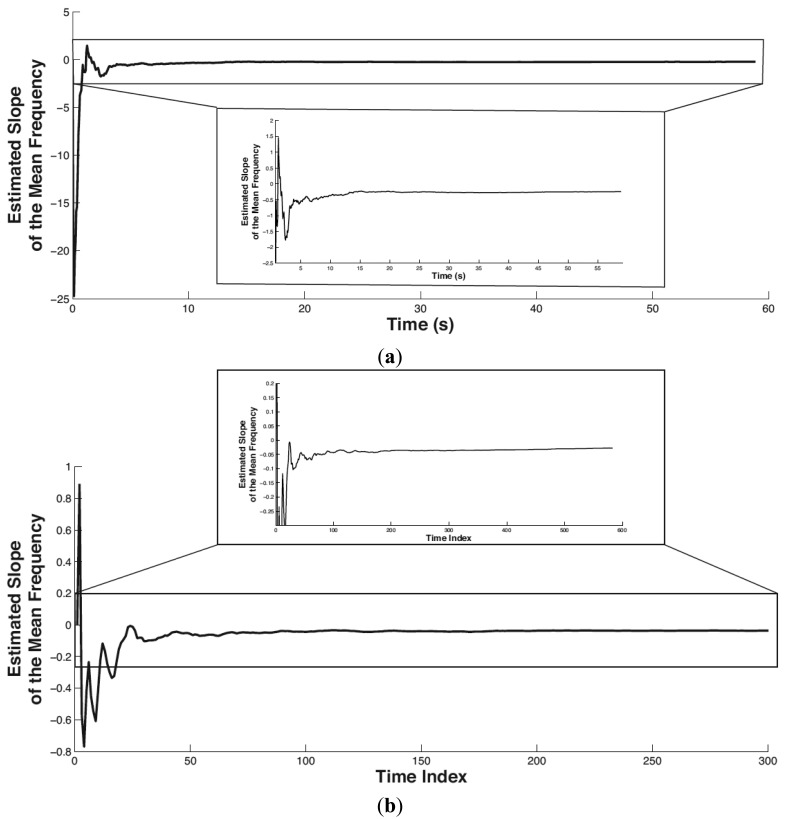
Tracked mean frequency trend of biceps brachii. (**a**) Experiment 1. (**b**) Experiment 2. In both Experiment 1 and Experiment 2, the tracked slope of the mean frequency variance converges to a negative value after a short period (around 5 s), indicating a linear decrease of the mean frequency with the muscle's working time.

In this paper, the setting of the forgetting factor is *λ* = 0.95, which means the previous localized muscular fatigue level information is partially used in calculating the current muscular fatigue level. The initial value of *φ̂_t_* and *p_t_* is *φ̂_t_*(0) = 0, *p_t_*(0) = 10,000. The tracked trend of the mean frequency of biceps brachii is shown in Figure 8 where (a) and (b) correspond to Experiment 1 and Experiment 2, respectively. In both subplots, the partial view of the mean frequency trend shown below is plotted by limiting the x-scale. The horizontal axis denotes time in seconds (or time index). The vertical axis denotes the tracked slope (*i.e.*, trend) of the mean frequency.

It is easy to see that the proposed method stabilizes in tracking the mean frequency trend within the initial 5 s. The slope of the mean frequency variance in Experiments 1 and 2 converges to −0.247 and −0.027, respectively. Both the slopes in Experiments 1 and 2 are negative, which is consistent with the scenario of physical fatigue. As the magnitude of time (in Experiment 1) is smaller than the time index (in Experiment 2) on the order of 10, correspondingly, the amount of the tracked slope in Experiment 1 is larger than that in Experiment 2 on the order of 10.

### 6.4. Localized and Overall Fatigue Levels

**Figure 9.**
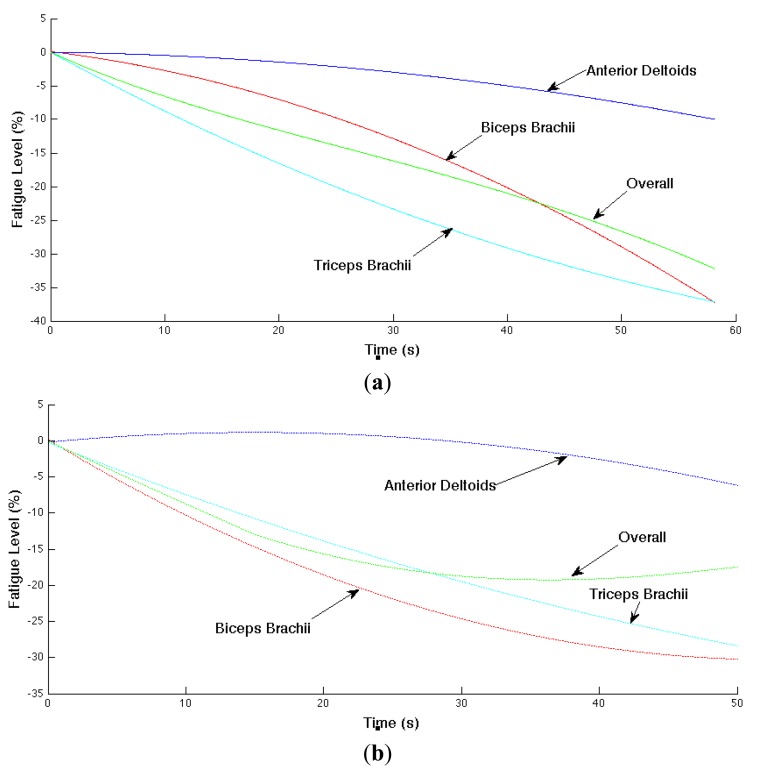
Overall fatigue level computed by localized fatigue levels of biceps brachii, anterior deltoids, and triceps brachii. (**a**) Experiment 1. (**b**) Experiment 2. The overall fatigue level starts around 0%, decreases gradually to −32% and −17%, in Experiments 1 and 2, respectively.

The localized muscular fatigue level is updated based on the mean frequency trend (*i.e.*, estimated slope parameter of the mean frequency). In addition, we smoothed the computed fatigue level for better result visualization. The muscular and overall fatigue level trajectories are shown in Figure 9 where (a) and (b) correspond the computed overall fatigue level and corresponding localized muscular fatigue levels in Experiments 1 and 2, respectively. The horizontal axis is time (or time index) and vertical axis is the computed fatigue level in negative percentages.

In Experiment 1 (shown in (a)), the localized muscular fatigue level of the biceps brachii, anterior deltoids and triceps brachiii starts from 0%, indicating a non-fatigue status, and decreases gradually to −37%, −9% and −37%, indicating a fatigue status, whereas the overall fatigue level provides a compromise of the localized muscular fatigue levels starting from 0% to −32%. Similarly, in Experiment 2 (shown in (b)), the localized fatigue level of the biceps brachii, anterior deltoids, triceps brachii and the overall fatigue level starts from 0%, decreases to −30%, −6%, −28%, −17%, respectively.

### 6.5. System Validation

The developed fatigue-tracking system was evaluated with two experiments involving 17 subjects (male group: 10 subjects; female group: 7 subjects). The detailed experiment setting was explained in Section 2.3. The subjects were quite diverse, originating from 11 countries. According to the BMI classification, the subject group covered underweight, normal weight, overweight and obesity categories.

The statistics of the fatigue levels is given in Table 2. In both experiments, the localized fatigue levels (corresponding with biceps brachii, anterior deltoids and triceps brachii) and overall fatigue level at the end of the experiments are listed. As the designed experiments targeted at monitoring fatigue scenario, all the fatigue levels are negative, which is consistent with the observed muscle-shaking scenario. Confirmed by the feedback from the subjects after the experiments, we approximately conclude that the more tired the subject feels, the bigger the overall fatigue level is. Besides, as seen in Table 2, the fatigue levels differ between subjects. However, if the subject had a relatively higher overall fatigue level in Experiment 1, he/she always had a relatively higher overall fatigue level in Experiment 2, correspondingly.

To evaluate the system from users' experience, we conducted a survey including seven questions as shown in Table 3. The questions cover the alertness, muscle fatigue feeling, calmness and comfort in both experiments. The rating starts from 1 to 5 corresponding with the least to the most intensity. Each subject is asked to complete the survey after the experiments.

**Figure 10.**
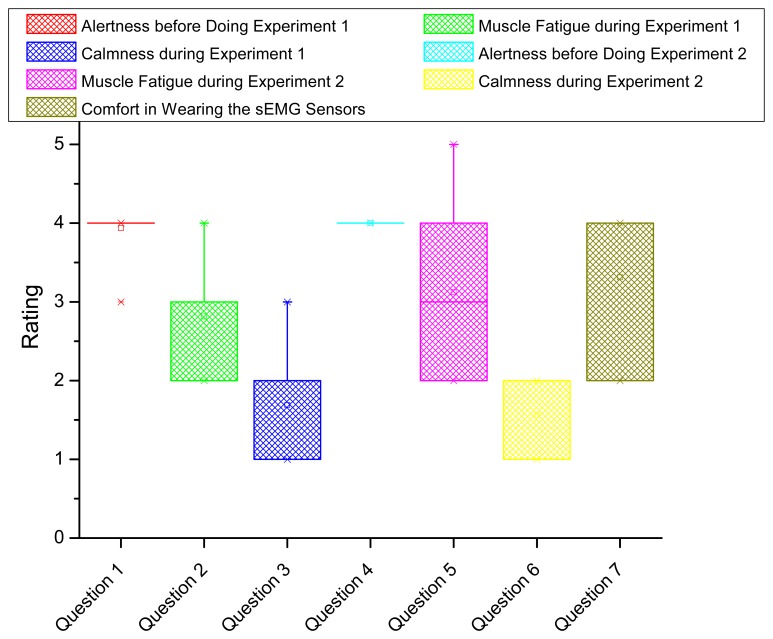
Statistics of the survey results regarding the fatigue experiments. Totally, 7 questions are proposed on the alertness, muscle fatigue feeling, calmness and comfort in the fatigue experiments.

The statistics of survey results is shown by box plot in Figure 10. The horizontal axis indicates the question index. The vertical axis shows the rating statistics. For each box (corresponding with one question), the upper and lower boundary represents the 25% and 75% of the interquartile range. The line and small square in the middle of the box indicates the median and mean value of the rating, respectively. Two small crosses locating above and below the box show the boundary of 1% and 99% of the rating values in the whole range. In Figure 10, we conclude the following results. First, nearly all the subjects are fully awake and alert before doing the experiments (from Questions 1 and 4), meaning their physiological status is proper for doing fatigue experiments. Second, during the fatigue experiments, the subjects averagely feel moderate fatigue whereas some of the subjects feel extreme fatigue in Experiment 1 and feel the worst fatigue in Experiment 2. All the subjects marked at least mild fatigue according to his/her feeling, which is consistent with the negative fatigue levels in Table 2. Overall, the subjects feel more fatigue in Experiment 2 compared in Experiment 1 (from Questions 2 and 5). Third, we compare the fatigue intensity change in Experiments 1 and 2 between the computed overall fatigue level and the subject's feeling where 73% coincidence is confirmed. Fourth, during the fatigue experiments, the subjects are in the condition between calm and slightly anxious (from Questions 3 and 6). Fifth, generally, the subjects feel the proposed fatigue-tracking system is acceptable in comfort (between moderately comfortable and very comfortable in Question 7).

## 7. Conclusions and Future Work

In this paper, we developed a wearable wireless system for tracking the status of fatigue in human movement. The proposed system is based on scientific electromyography and kinesiology studies, which show that the mean frequency decreases with the increase of the fatigue intensity. According to previous work, we assume that the decrease of mean frequency satisfies a linear relation with working time of a muscle under fatigue. We then used a rigorous statistical analysis to prove this assumption, upon which the definition of localized muscular fatigue level is based on. We then tracked the localized muscular fatigue levels by updating the parameters with the most current measurements by considering the fatigue process as a dynamic process. Furthermore, the overall fatigue level corresponding to a human movement was computed by fusing different localized muscular fatigue levels together. Finally, the developed fatigue tracking system was tested and verified with two fatigue experiments involving 17 subjects. In the proposed method, the setting of the “forgetting factor” and fatigue level fusion coefficient might vary according to different muscle types and practical applications. This could be a limitation to the application of our method. Nonetheless, our future work is to clarify this parameter setting issue.

## Figures and Tables

**Table 1.** Statistical Analysis of Variance (ANOVA).

**Subject**		**Gender Group**									
No.		m1	m2	m3	m4	m5	m6	m7	m8	m9	m10

F	F	296.625	677.604	59.511	18.423	8.032	205.884	57.706	659.796	17.993	482.152
test	*p*	0.000	0.000	0.000	0.000	0.005	0.000	0.000	0.000	0.000	0.000

No.		f1	f2	f3	f4	f5	f6	f7			

F	F	968.508	40.212	4.174	438.636	523.176	134.794	1310.249			
test	*p*	0.000	0.000	0.041	0.000	0.000	0.000	0.000			

**Table 2.** Statistics of the fatigue levels.

**Subject**	**No.**	**Fatigue Levels in Experiment 1**				**Fatigue Levels in Experiment 2**			

		**Biceps** **Brachii**	**Anterior** **Deltoids**	**Triceps** **Brachii**	**Overall**	**Biceps** **Brachii**	**Anterior** **Deltoids**	**Triceps** **Brachii**	**Overall**
Male Group	m1	−9%	−13%	−8%	−10%	−9%	−24%	−20%	−20%
	m2	−8%	−25%	−39%	−31%	−21%	-25%	−42%	−32%
	m3	−21%	−19%	−31%	−25%	−8%	−19%	−28%	−22%
	m4	−13%	−18%	−21%	−18%	−10%	−40%	−24%	−31%
	m5	−14%	−6%	−15%	−13%	−16%	−15%	−27%	−21%
	m6	−12%	−23%	−11%	−17%	−5%	−15%	−29%	−22%
	m7	−14%	−21%	−15%	−17%	−10%	−17%	−56%	−42%
	m8	−8%	−20%	−13%	−15%	−14%	−25%	−46%	−35%
	m9	−11%	−11%	−13%	−12%	−14%	−11%	−36%	−26%
	m10	−10%	−21%	−16%	−17%	−4%	−24%	−8%	−18%

Female Group	f1	−7%	−28%	−32%	−28%	−31%	−15%	−47%	−37%
	f2	−22%	−5%	−36%	−29%	−33%	−15%	−24%	−26%
	f3	−37%	−9%	−37%	−32%	−30%	−6%	−28%	−17%
	f4	−3%	−24%	−39%	−32%	−10%	−4%	−43%	−34%
	f5	−14%	−28%	−14%	−21%	−13%	−19%	−14%	−16%
	f6	−4%	−18%	−9%	−14%	−3%	−5%	−11%	−8%
	f7	−10%	−39%	−16%	−29%	−12%	−5%	−35%	−27%

**Table 3.** Survey of fatigue experiments.

**Questions**	**1**	**2**	**3**	**4**	**5**
Q1: Alertness before Doing Experiment 1	Deeply asleep	Lightly asleep	Drowsy	Fully awake and alert	Hyper-alert
Q2: Muscle Fatigue Feeling during Experiment 1	No fatigue	Mild fatigue	Moderate fatigue	Extreme fatigue	The worst fatigue
Q3: Calmness during Experiment 1	Calm	Slightly anxious	Anxious	Very anxious	Panicky
Q4: Alertness before Doing Experiment 2	Deeply asleep	Lightly asleep	Drowsy	Fully awake and alert	Hyper-alert
Q5: Muscle Fatigue Feeling during Experiment 2	No fatigue	Mild fatigue	Moderate fatigue	Extreme fatigue	The worst fatigue
Q6: Calmness during Experiment 2	Calm	Slightly anxious	Anxious	Very anxious	Panicky
Q7: Comfort in Wearing the Fatigue-Tracking System	Not comfortable at all	Mildly confortable	Moderately comfortable	Very comfortable	Extremely comfortable
